# Draft Genome Sequence of Halomonas elongata MH25661 Isolated from a Saline Creek in the Andes of Peru

**DOI:** 10.1128/MRA.00934-18

**Published:** 2019-01-03

**Authors:** J. Ilucion Mamani, Kevin B. Pacheco, Paola Elorrieta, Pedro Romoacca, Hugo Castelan, Sonia Davila, Jose L. Sierra, Maria A. Quispe-Ricalde

**Affiliations:** a Departamento de Biología, Facultad de Ciencias, Universidad Nacional de San Antonio Abad del Cusco, Cusco, Peru; b Departamento de Farmacia y Bioquímica, Facultad de Ciencias de la Salud, Universidad Nacional de San Antonio Abad del Cusco, Cusco, Peru; c Centro de Investigaciones en Dinámica Celular, Instituto de Investigación en Ciencias Básicas y Aplicadas, Universidad Autónoma del Estado de Morelos, Cuernavaca, Mexico; d Escuela de Postgrado, Universidad Nacional de San Antonio Abad del Cusco, Cusco, Peru; Loyola University Chicago

## Abstract

Halomonas elongata strain MH25661, a moderate halophile, was isolated from a saline creek in the Andes of Peru. Genome annotation analyses revealed genes that respond to osmotic stress and oxidative stress, genes that are involved in sporulation, and genes that are involved in transport systems and resistance to antibiotics and toxic compounds.

## ANNOUNCEMENT

Strain MH25661, which corresponds to the Halomonas elongata species, belongs to the class *Gammaproteobacteria* (1). The genus *Halomonas* is the largest member within the family *Halomonadaceae* (2). H. elongata is a moderate halophile, aerobic, Gram-negative, rod-shaped bacterium (1), which has the ability to grow in saline environments such as hypersaline lakes, seas, hydrothermal vents, and solar salt facilities (2). The members of the *Halomonas* genus are distributed in a range of depths from 2,000 m under the sea (3) to 4,600 m above sea level in salty Andean lakes (4), growing at high levels of UV radiation and with a small amount of nutrients and in the presence of heavy metals (4).

The strain MH25661 of H. elongata was obtained from a saline creek in the Huanoquite district of the province of Paruro, department of Cusco, Peru (13°40.511′S, 72°01.384′W). The saline source is located 3,255 m above sea level, originating underground in the Andes in the Southwest of Peru, and contains 17% NaCl. The isolation of the MH25661 strain was carried out through filtering (0.22 μm) the water from the saline creek and culturing the filter in sea water (SW) medium with 25% NaCl (wt/vol) (5) for 96 h at 37°C. The DNA was isolated with the GenElute genomic DNA bacteria kit (Sigma-Aldrich). A paired-end library with average insert size of 300 bp was prepared with the Nextera XT DNA library kit (Illumina Inc.) using 1 ng of DNA as the initial genomic sample. The sequencing was executed on a MiSeq System (Illumina Inc.; GeneLab del Perú SAC), with 375,660 reading sequences and a read size of 2 to 231 bp.

Assembly of the genome was made with the A5 assembler version MiSeq Linux 20160825 (6). The quality analysis of the assembly was carried out with the QUAST program (7). The genome contains 3,552,403 bp in 117 contigs (*N*_50_, 82,628 bp), and the longest is 210,370 bp. The maximum 21× coverage was obtained. In both cases, default settings for the software programs were used.

The functional annotation was performed with the Rapid Annotation using Subsystem Technology (RAST) server from contigs (8) (http://rast.nmpdr.org), which produced 3,247 protein-coding sequences. The genome draft has a G+C content of 64.3%, which is similar to other *Halomonas* species. In this study, the 16S rRNA gene of H. elongata was obtained with RAST annotation, and the similarity with a BLAST search was 99%.

The functional analysis showed 463 subsystems, which indicated important genes, among them the ectoine gene, which is responsible for the osmotic stress response; the glutathione gene, which is responsible for oxidative stress response; the CspA proteins, which are responsible for cells adapting to cold temperatures; the hemin and siderophores (aerobactin) transport systems, which are are responsible for the assimilation and metabolism of metals; and numerous genes in charge of transport systems, as aromatic compounds. In addition, this strain contains several genes involved in resistance to antibiotics and toxic compounds.

### Data availability.

This whole-genome project for H. elongata strain MH25661 has been deposited at GenBank under the accession number QJUB00000000. The version described in this paper is accession number QJUB01000000. The SRA accession number is SRR7973874.
